# Vertically Transmitted Gut Bacteria and Nutrition Influence the Immunity and Fitness of *Bactrocera dorsalis* Larvae

**DOI:** 10.3389/fmicb.2020.596352

**Published:** 2020-10-30

**Authors:** Babar Hassan, Junaid Ali Siddiqui, Yijuan Xu

**Affiliations:** Laboratory of Quarantine and Invasive Pests, Department of Entomology, South China Agricultural University, Guangzhou, China

**Keywords:** symbiotic bacteria, diet diversity, protein, carbohydrates, immunity, survival, fitness

## Abstract

Symbiotic bacterial communities that colonize the digestive tract of tephritid fruit flies interact with nutrient intake to improve the flies’ fitness and immunity. Some bacterial species consistently inhabit the tephritid guts and are transmitted to the next generation vertically. These species contribute significantly to some aspects of their host’s physiology. In the current study, we examined the role of four vertically transmitted bacteria (*Citrobacter*, *Enterobacter*, *Klebsiella*, and *Providencia*) on the fitness parameters and immunity of *Bactrocera dorsalis* larvae that were fed a nutritionally manipulated diet. For this purpose, eggs were collected from axenic, gnotobiotic, and symbiotic adult flies, and larvae were reared on four types of diets in which carbohydrate and/or protein contents were reduced and then compared with larvae raised on a control diet. The diet and bacterial interactions significantly affected the fitness and immunity of *B. dorsalis*. Larvae of axenic flies grew slower and displayed weaker immune-based responses (PO activity, antibacterial activity, survival) than larvae of gnotobiotic and symbiotic flies. Overall, larvae reared on the low-protein diet grew slower than those reared on the control or low-carbohydrate diets. Survival, PO activity, and antibacterial activity were significantly lower in the hemolymph of larvae reared on low-protein diets. Our results also revealed that the levels of hemolymph protein, glucose, trehalose, and triglyceride in larvae from axenic flies were significantly lower than those in larvae of the symbiotic group after they fed on most of the tested diets. These results strongly infer that diet and vertically transmitted bacteria are both essential contributors to the fitness and immunity of *B. dorsalis*.

## Introduction

Insect immune function requires high energy expenditure for optimal performance while fighting pathogenic or parasitic infection. There are trade-offs in the allocation of resources between immune functions and other fitness components of insects. Previous studies have shown that the nutritional state of the insect and the quality of its food have substantial effects on life-history traits and immune function (Lee et al., 2008; Cotter et al., 2011; Ponton et al., 2011; Mason et al., 2014). Insects subjected to limitations in food availability balance their energy budget among life-history traits and immune function. Restriction of food or low-quality diets can compromise the immune response of insects (Lee et al., 2008).

The insect immune system has several components that react differently to nutrients; therefore, different levels of food restrictions or quality produce different immune response patterns (Ponton et al., 2011; Krams et al., 2015). Intracellular immune signaling pathways are interconnected with nutrient signaling pathways in insects that make interactions between food deprivation and immunity very complex (Ponton et al., 2011, 2013; Adamo et al., 2016). Therefore, the immune system does not always gradually decline as resources decline. For instance, food deficiency in *Drosophila melanogaster* leads to an increase in AMP gene expression even in the absence of pathogens (Becker et al., 2010). Macronutrients of the diet mediate the physiological functions of the immune system in insects. Several studies have been conducted to determine the effects of macronutrients such as protein and carbohydrates on insect fitness traits and immunity (Scriber and Slansky, 1981; Lee et al., 2008; Srygley et al., 2009; Cotter et al., 2011; Le Gall and Behmer, 2014).

Insects harbor diverse bacterial communities in their digestive tract, and most of them are benign or beneficial to their hosts (Dillon and Dillon, 2004). These mutualistic bacteria are essential for host behavior manipulation, nutrition, digestion, detoxification of harmful chemicals, and adaptation to different environments (Feldhaar, 2011; Douglas, 2015). Gut bacterial communities develop complex interactions with their host niches and strive with opportunistic germs for nutrients and attachment sites on epithelial surfaces of the gut. Therefore, regulation of bacterial communities in the gut is essential to avoid uncontrolled proliferation (Oliver et al., 2006; Krams et al., 2017). Insects have evolved chemical, nutritional, and immunological mechanisms to ensure the maintenance of bacterial communities necessary to meet their needs (Huang and Douglas, 2015). Immunological mechanisms include the insect immune system that recognizes and regulates bacterial mutualists in the gut (Login et al., 2011). Manipulation of the microbiota, either via antibiotic treatment or microbiota reconstitution, provides critical evidence for the role of the microbiota in the immune system (Mazmanian et al., 2005; Sekirov et al., 2008). Removal or alteration of the gut bacterial communities of the host alters host susceptibility to enteric infection and causes several diseases, such as gastroenteritis, metabolic imbalance, and inflammatory bowel disease, due to failure of the immune system (Garrett et al., 2010; Krams et al., 2017). The gut microbiota can not only regulate the local gut immune system but also have a profound influence on systemic immune responses.

*Bactrocera dorsalis* (Diptera: Tephritidae) is a menace to the horticultural industry around the world (Benelli et al., 2014). Like other insects, *B. dorsalis* and other members of the family Tephritidae have close relationships with symbiotic bacteria that have been isolated from the digestive systems, and the significant roles they play are extensively recognized (Lauzon, 2003). Bacterial communities in the digestive tract of fruit flies influence their fitness and life-history traits by contributing to host metabolism (Cai et al., 2018; Khan et al., 2019). These have been reported to synthesize essential amino acids that do not otherwise occur in the diet and increase protein synthesis and female fecundity (Miyazaki et al., 1968; Tsiropoulos, 1984; Behar et al., 2005; Ben-Yosef et al., 2010, 2014). Fruit fly gut bacterial communities have also been reported to facilitate nutrient uptake, strengthen mating competitiveness, defend against natural enemies, prolong or shorten the host lifespan and help detoxify plant allelochemicals and pesticides (Dillon and Dillon, 2004; Behar et al., 2008b; Ben-Yosef et al., 2014; Cheng et al., 2017; Akami et al., 2019).

Tephritid fruit flies acquire a variety of microorganisms through horizontal and vertical transmission. Vertically transmitted bacteria consistently inhabit the tephritid gut and significantly contribute to some aspects of their host’s physiology (Wang A. et al., 2014; Wang H. et al., 2014). These are transferred to offspring through contamination of the egg surface and deposition of bacterial capsules on eggs or transovarial transmission (Behar et al., 2008a; Lauzon et al., 2009; Szklarzewicz and Michalik, 2017). The Enterobacteriaceae family is the dominant family in the bacterial community inhabiting the reproductive system of *B. dorsalis*. *Citrobacter*, *Klebsiella*, *Providencia*, and *Enterobacter* species belong to the Enterobacteriaceae family and dominate the bacteria that are transferred vertically to the eggs, the fruit (during oviposition), and finally, the offspring (Behar et al., 2008a; Lauzon et al., 2009; Wang et al., 2011; Aharon et al., 2013; Wang A. et al., 2014; Wang H. et al., 2014; Guo et al., 2017).

Sterile insect technique (SIT) offers a promising potential strategy for the control of *B. dorsalis* (Ali et al., 2017). Several factors can directly affect the quality of the flies, but the artificial diet is of crucial importance for mass-reared fruit flies (Moadeli et al., 2018). Therefore, to increase the SIT’s efficiency, studies have focused on improving artificial diets (Hou et al., 2020). Recent studies showed that laboratory rearing of tephritid fruit flies on the artificial diets leads to the loss of certain bacterial communities in the gut. Many other transiently acquired bacteria replace some essential gut bacteria and become the reason for laboratory rearing inefficiency for these tephritid flies (Sacchetti et al., 2008; Kounatidis et al., 2009). Therefore, it is crucial to improve artificial diets, which may be achieved with bacterial enrichment of laboratory diets. Several studies suggest the use of crushed guts of adult flies as larval additives. However, this method is very labor intensive (Koskinioti et al., 2020); therefore, feeding the targeted cultivable bacterial isolates originating from adult *B. dorsalis* to benefit artificial larval rearing can be used to improve oriental fruit fly larval rearing for SIT programs.

To date, however, little is known about how members of the vertically transmitted gut microbiota interact to shape *B. dorsalis* development, fitness, and immunity. Protein and carbohydrates are the main nutritional macronutrients in diets used for the rearing of fruit flies, providing vitamins, lipids, and minerals to support the development of insects (Chan et al., 1990; Chang et al., 2000). These macronutrients mediate normal physiological functioning in *B. dorsalis*. In the present study, we studied the effects of some members of the vertically transmitted gut microbiota and diet on the fitness traits and immunity of *B. dorsalis* larvae. The concentrations of some important hemolymph metabolites, including protein, glucose, triglycerides (TAGs), and trehalose, which can accurately indicate the nutrition and metabolic status of hosts, were measured to reveal the physiological consequences of the gut microbiota and diet on *B. dorsalis*. This study greatly improves our understanding of how diet diversity and the manipulation of gut bacteria affect the fitness of oriental fruit flies and may have significant implications for SIT programs.

## Materials and Methods

### Insects

All experiments were performed using a laboratory strain of *B. dorsalis* originally collected from Guangzhou, China, in May 2015. The larvae of *B. dorsalis* were fed a banana and maize-based artificial diet containing 150 g of cornflour, 150 g of banana, 0.6 g of sodium benzoate, 30 g of yeast, 30 g of sucrose, 30 g of paper towel, 1.2 mL of hydrochloric acid, and 300 mL of water. The adults were reared with water and a 1:3 mixture of yeast hydrolysate and sucrose (Cheng et al., 2017). Experimental conditions consisted of a temperature of 25 ± 1°C and a 16:8 h light: dark photoperiod with 60–70% relative humidity.

### Isolation and Identification of Gut Bacteria

Total gut bacteria of flies were identified previously (Cheng et al., 2017). For the preparation of gnotobiotic flies, four vertically transmitted bacteria of the family Enterobacteriaceae were selected based upon their high abundance in the *B. dorsalis* guts and their role in flies according to the literature. These strains were *Citrobacter* spp., *Klebsiella* spp., *Providencia* spp., and *Enterobacter* spp. The approximate proportions of these four bacterial genera in the family Enterobacteriaceae were 43, 10, 31, and 4.29% (Cheng et al., 2017). For the isolation and identification of these bacterial isolates, 3-day-old male and female adult flies were selected, washed with ethanol (70%) for at least 3 min to remove surface bacteria and then washed with sterile phosphate-buffered saline (PBS). The guts of flies aseptically dissected under a stereomicroscope were transferred into sterile centrifuge tubes containing 200 μl of PBS (1X, pH 7.4). The guts were then ground with sterile pestles and homogenized. One hundred microliters of the fluid was diluted up to 10^–6^ and plated on Luria-Bertani (LB) agar plates [LB broth (tryptone, yeast extract NaCl): 2.1 g, agar: 1.5 g, water: 100 ml] that were incubated for 24–48 h at 28°C. Based on colony morphology, 10–15 colonies were selected from plates and further purified by sub-culturing. The pure cultures were inoculated into LB liquid (LB broth: 2.1 g, water: 100 ml) cultures and stored in 25% glycerol solution at −80°C. For identification, bacteria were collected from pure cultures for the extraction of genomic DNA using a Bacterial Genome DNA Extraction Kit (Tiangen Biotech Co., Ltd., Beijing, China) according to the manufacturer’s instructions. 16S rDNA amplification was performed using universal primers (27F/1459R), and the products were sequenced. For amplification and sequencing (Sanger sequencing), we used services provided by GENEWIZ^1^. The sequences were subjected to a BLAST search against the NCBI database for sequence homology analysis (Supplementary Table S1).

### Microbiota Manipulation and Collection of Eggs

A detailed pictorial representation of the experiments is given in Supplementary Figure S1. Eggs were collected from symbiotic, axenic, and gnotobiotic adult flies produced from the laboratory-established colony by following previously published methods (Cheng et al., 2017; Akami et al., 2019) with some modification. Briefly, 200 newly emerged adult males and females were housed in a cage at equal proportions for the collection of eggs. Collected eggs were placed on banana and maize-based artificial diets until the third instar of larvae. Larvae were allowed to pupate in sterile sand, and pupae were collected (4 days old) from the sand and housed in the adult cage. Emerging adults were divided into three groups, each having 200 male and female flies (1:1). The symbiotic group received an autoclaved regular adult diet (yeast hydrolysate: sugar and water) without antibiotics provided *ad libitum* until they started laying eggs. The axenic group was reared on an autoclaved regular diet along with antibiotics (penicillin 100 μg/ml, streptomycin 100 μg/ml, gentamicin 150 μg/ml, rifampicin 150 μg/ml, tetracycline 50 μg/ml) until they started laying eggs. The gnotobiotic group was fed an autoclaved regular diet and antibiotics for the first 72 h to remove their gut bacteria. These flies were fed a regular diet along with selected bacterial strains until they started laying eggs. *Citrobacter, Klebsiella, Providencia*, and *Enterobacter* species previously isolated from the guts of *B. dorsalis* were supplemented at an OD600 of 0.8 in the regular diet for at least 4 days. The bacterial isolates were cultured individually in LB broth and harvested by centrifugation. They were later washed twice with sterile distilled water, resuspended again in sterile distilled water and then added to the diet.

The axenic status of flies was confirmed for gnotobiotic flies before they were fed bacterial strains. For this purpose, 10 flies were removed from the rearing cage and washed with ethanol (70%) and sterile water. These were dissected aseptically, the guts were homogenized, and the homogenates were serially diluted up to a 10^–6^ dilution in PBS. A total of 100 μl of each dilution was spread onto LB agar plates and incubated at 28°C for 48 h. Then, the colony-forming units (CFU) resulting from the bacterial colonies on each plate were averaged and analyzed. On days 7–8, the status of flies was again confirmed by dissecting ten flies from each group, and bacteria were cultured on LB agar plates to confirm the differences in the bacterial populations of the three groups of flies.

Eggs were collected from the three groups using a sterile brush. Eggs collected from axenic flies were further sterilized by washing them twice in 0.5% chlorite liquid bleach for 5 min, followed by one wash in 70% ethanol for 2 min and three washes in Milli-Q water. However, eggs collected from gnotobiotic and symbiotic flies were directly transferred to the diet in a sterile environment using a sterile brush.

Twenty-five eggs collected from each group were crushed in sterile PBS in a sterile environment and plated on LB agar plates for 24 h at 28°C to confirm the differences in bacterial colonies in each group. Moreover, the size and viability of eggs collected from axenic, symbiotic, and gnotobiotic flies were also determined. For egg viability, a total of 150 eggs from each group (50 eggs/replicate) were placed in sterile petri plates containing sterile filter paper and sterile saline solution, and the number of hatched larvae was counted under a microscope after 2 days.

### Preparation of Diet

A liquid gel-based larval diet was prepared as per the method of Khan et al. (2019). Four experimental diets were designed differing in their protein and carbohydrate contents. The protein content was manipulated using a mix of soy protein and baking yeast. The carbohydrate content was manipulated using sucrose only. The main component in soy bran is fiber (72 g/100 g), and it has almost equal amounts of protein and carbohydrates, ≈ 6–7.5 g; therefore, it was kept constant, similar to other ingredients (Table 1). The carbohydrate and protein contents of the diets were 20% or 50% of the control amounts in separate treatments. One treatment included a 50% reduction in both carbohydrate and protein components relative to their levels in the control treatment. Most of the larvae feeding on the diet in which protein contents were 20% did not survive after 3–4 days of hatching, and few with very small size did not molt to pupae until 12 days. Therefore, this treatment was not considered for further experiments. For diet preparation, all ingredients were weighed and mixed in a blender with half the water until the ingredients were sufficiently homogenous. Agar was then mixed with the rest of the water and heated for 5 min in a microwave. Heated agar was added to the ingredients in the blender, and they were mixed again until homogenous. The diet was poured into a disposable round bowl (dia: 9.5 cm, Guangzhou Jianxin Plastic Products Factory) or falcon tubes (50 ml) immediately and left to cool at room temperature. Fifteen treatments (5 levels of diet × 3 levels of gut bacteria) were used for further experiments.

**TABLE 1:** Components used to prepare the artificial diet (1 L) for the rearing of *B. dorsalis* larvae.

**Ingredients**	**Diet types**					
	**Control**	**P50**	**P20**	**C50**	**C20**	**PC50**
Baking yeast (g)	37.70	18.85	7.54	37.70	37.70	18.85
Sugar (g)	89.60	89.60	89.60	44.80	17.92	44.80
Soy protein (g)	75.10	37.55	15.02	75.10	75.10	37.55
Soy bran (g)	38.60	38.60	38.60	38.60	38.60	38.60
Citric acid (g)	17.60	17.60	17.60	17.60	17.60	17.60
Sodium benzoate (g)	2.90	2.90	2.90	2.90	2.90	2.90
Milli-Q water (ml)	738.50	738.50	738.50	738.50	738.50	738.50
Agar (g)	3.33	3.33	3.33	3.33	3.33	3.33

### Effect of Diet and Gut Bacterial Manipulation on Development

For this experiment, we followed the method adopted by Morimoto et al. (2019) with the following modifications. Approximately 500 eggs (two petri dishes, each having 250 eggs) were allowed to hatch on moist sterile filter paper in covered petri dishes in a sterile environment (Weldon et al., 2019). Using a dissecting microscope, a total of 160 newly hatched larvae for each treatment (40 larvae/replicate) were placed in the middle of falcon tubes (50 ml) containing a gel-based diet (30 ml) using a sterile brush under sterile conditions. For the preparation of diet tubes, the diet was poured while warm, and tubes were tilted until the diet material set to generate more diet surface area for the larvae. Excess moisture was allowed to evaporate under sterile conditions before sealing tubes. The development of flies on four different breeding diets was measured. For all groups, we monitored the time course of larval and pupal development, larvae and pupal weight, and adult eclosion rate. The weights of larvae (18–20 larvae) were measured when larvae started coming out from the diet near the lids of falcon tubes. Pupal weight (15–20 pupae) was measured after 4 days of pupation. The method of pupae collection was the same as that described by Morimoto et al. (2019). The adult eclosion rate was determined by counting the number of adults that emerged from all harvested pupae from each group separately.

### Method of Bacterial Infection and Collection of Hemolymph

For each treatment, 250 newly hatched larvae (50 larvae/replicate) were released using a sterile brush in a sterile environment on the top of the strip of wet filter paper placed in the middle of the diet in the bowls. Larvae were allowed to grow up to the third instar (4–5-day-old larvae) and then used for further experiments.

We used two bacteria, *Escherichia coli* and *Staphylococcus aureus*, as antigens to challenge *B. dorsalis* larvae (Shi et al., 2015). The two bacteria were grown to a stationary phase in LB broth at 37°C before each infection day. On the day of infection, stationary cultures were diluted in sterile LB broth to A600 = 1.0. Larvae (4 days old) were infected by pricking with needles (0.10 mm) that had been dipped in both diluted bacterial suspensions in physiological saline (Unckless et al., 2015). The larvae pricked with a sterilized pin dipped in saline solution or undipped represented the controls. The bacterial load in infected and uninfected larvae was determined by culturing on LB plates 24 h after infection. For hemolymph collection, each larva was washed with sterile water to remove excrement and food particles and then anesthetized for 3–5 min on ice after 24 h of infection. Subsequently, its epidermis was pierced by a fine sharp, sterile needle for collecting hemolymph into a labeled sterile clean microcentrifuge tube (1.5 ml).

### Effect of Diet and Gut Bacterial Manipulation on Immune Function

Collected hemolymph was used to determine the effects of food quality and gut bacterial manipulation on two immune function traits: hemolymph phenoloxidase (PO) activity and antibacterial activity. For the measurement of PO activity, we followed the method adopted by Lee et al. (2008) with some modifications. Briefly, 8 μL of hemolymph was added to 400 μL of ice−cold PBS (pH 7.4) in a plastic Eppendorf tube and then centrifuged at 8000 *g* for 5 min. The supernatant was extracted and used for PO activity immediately. A 100 μL aliquot of 10 mm L-Dopa (substrate) was added to 100 μL of supernatant. The mixture’s absorbance was measured at 490 nm on a microplate reader after 20 min of incubation at 25°C. A bicinchoninic acid (BCA) protein assay kit was used to measure the protein contents of hemolymph. The experiment was repeated three times using the hemolymph of different larvae of the same treatment group.

Antibacterial activity of hemolymph collected from *B. dorsalis* larvae was measured in a sterile 96-well plate with a final volume of 140 μl against *E. coli* cells that were cultured overnight as described above. An aliquot of 100 μl of bacterial culture or sterilized PBS or tetracycline solution (1 mg/ml) was added to 40 μl of hemolymph samples. Then, plates were incubated at 30°C for 12 h. The growth of bacteria was measured as the cell concentration, which was determined by the absorbance value at 600 nm using a microplate reader (Shi et al., 2015). The experiment was repeated three times using the hemolymph of different larvae of the same treatment group.

### Bacterial Septic Infection and Measurement of Survival Rate

The post infection survival of insects infected with *E. coli* and *S. aureus* was calculated. For this purpose, 20 insects for each treatment were infected with both bacteria, as described above. The mortality of larvae was observed daily until pupation or death of all individuals.

### Nutritional Indices in *B. dorsalis* Larvae

Specific metabolite profiles might be associated with changes in immunity. Therefore, we assayed nutritional indices in larvae reared on each diet. Third-instar larvae were used to collect the hemolymph for the quantification of nutritional indices, including TAG, protein, trehalose, and glucose concentrations. For hemolymph collection, each larva was washed with sterile water to remove excrement and food particles and then anesthetized for 3–5 min on ice. Subsequently, its epidermis was pierced by a fine sharp needle for collecting hemolymph into a labeled clean microcentrifuge tube (1.5 ml) along with 2 μl of 0.2% phenylthiourea (PTU) to inhibit hemolymph coagulation. The protein content of each sample was analyzed with a BCA protein assay kit (Tiangen Biotech Co., Ltd., Beijing, China). Glucose was measured using a Glucose Measurement Kit (Shanghai Rongsheng Biology Pharmaceutical Co., Ltd., China). TAG content was determined with a TAG assay kit (Zhejiang Dongou Diagnostic Products Co., Ltd., China). The concentration of trehalose was quantified with a trehalose assay kit (Megazyme Bray, Co., Wicklow, Ireland) by following the manufacturer’s instructions (Ridley et al., 2012; Unckless et al., 2015; Habineza et al., 2019).

### Statistical Analysis

Except as noted, all analyses were carried out using jamovi (version 1.2.22) for Windows. The normality of the data was assessed using the Shapiro–Wilk test. A general linear model (GLM) was used to analyze the test data and was followed by Tukey’s multiple comparison test. Correlation analysis was performed where needed. The Kruskal–Wallis *H* test was used to analyze the differences in CFUs in each group, and survival data were analyzed using a Kaplan–Meier survival test followed by the log-rank (Mantel–Cox) test in GraphPad Prism version 7.00 for Windows (GraphPad Software, La Jolla, CA, United States). Statistical significance was defined as *p* < 0.05.

## Results

### Generation of Axenic and Gnotobiotic Flies

Culture-dependent validations confirmed that the highest quantity of bacterial colonies was found in the guts of symbiotic flies, followed by gnotobiotic flies. Only one CFU was found in all plates cultured with guts of axenic flies (*H* = 26.0; *p* < 0.0001; Supplementary Figures S2a,b). This means that the oral antibiotic feeding of adult flies successfully removed the gut bacteria. Similarly, oral inoculation of adult axenic flies with five bacterial isolates resulted in successful colonization of the gut. The status of bacteria on eggs collected from the three groups was also confirmed by the LB agar plate detection method. No bacterial colony was detected on eggs collected from axenic flies. Eggs collected from symbiotic flies showed the highest number of colonies, followed by eggs of gnotobiotic flies (*H* = 13.0; *p* < 0.0001; Supplementary Figure S2c).

The size and viability of eggs collected from axenic, symbiotic, and gnotobiotic flies were also determined. Eggs collected from the three groups were of similar length (*H* = 2.95; *p* = 0.22) and width (*H* = 0.54; *p* = 0.74) (Supplementary Figures S3a,b). No significant difference was observed in hatching success (%) (*H* = 1.51; *p* = 0.54) of dechorionated eggs from axenic flies and non-dechorionated eggs of symbiotic and gnotobiotic flies (Supplementary Figure S3c). Similarly, the length of newly emerged larvae of each of the three groups of flies differed non-significantly (*H* = 0.92; 0.63) (Supplementary Figure S3d).

### Effect of Diet and Gut Bacterial Manipulation on the Development of *B. dorsalis*

**FIGURE 1:**
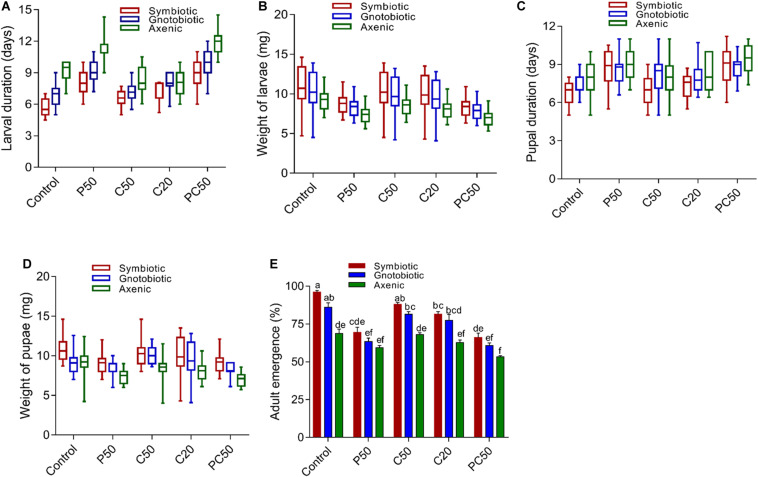
Effect of diet and gut bacterial manipulation on the development of *B. dorsalis* larvae: larval development duration **(A)**, larval weight **(B)**, pupal development duration **(C)**, pupal weight **(D)**, and adult eclosion rate **(E)**.

Gut bacterial manipulation significantly affected the larval development duration and weight of larvae. The period of larval development from axenic flies significantly increased compared to that of larvae from symbiotic and gnotobiotic flies irrespective of diet type (*F*_2_,_285_ = 129.17; *p* < 0.001). However, diet (*F*_4_,_285_ = 110.59; *p* < 0.001) and the interaction between diet type × fly groups (*F*_8_,_285_ = 5.50; *p* < 0.001) had significant effects. The time required for larval development when larvae had been reared on the P50 and PC50 diets was significantly greater than that of larvae on the control and C50 diets. The larval development time for the C20 diet was intermediate (Figure 1A). The larval development period of gnotobiotic flies was longer than that of symbiotic flies but shorter than that of larvae from axenic flies (9.65 days), showing that reintroduction of bacteria in flies has a significant effect on the larval development period. However, the larval weights from gnotobiotic and symbiotic flies were similar and higher than that for larvae from axenic flies (*F*_2_,_285_ = 26.58; *p* < 0.001). The weights of larvae reared with PC50 or P50 were less than those of the flies reared on the other diets, including the control (*F*_4_,_285_ = 22.08; *p* < 0.001) (Figure 1B). For larval weight, the diet type × fly group interaction was not significant (*F*_8_,_285_ = 0.24; *p* = 0.98).

Unlike the larval development period, the pupal development times of axenic and gnotobiotic larvae were similar and higher than that of symbiotic larvae (*F*_2_,_285_ = 10.12; *p* < 0.001). Compared to the rest of the diet treatments, the PC50 and P50 diets increased the pupal developmental time (*F*_4_,_285_ = 19.19; *p* < 0.001) (Figure 1C). However, the diet type × fly group interaction effect was non-significant (*F*_8_,_285_ = 1.29; *p* = 0.24). Pupal weight was reduced in axenic flies compared to symbiotic and gnotobiotic flies. The reintroduction of bacteria in the flies increased the pupal weight, but it differed significantly from that of symbiotic flies (*F*_2_,_285_ = 42.47; *p* < 0.001). Similarly, a reduction in the protein content of the diet (PC50, P50) also reduced pupal weight compared to those for the other diets (*F*_4_,_285_ = 16.05; *p* < 0.001) (Figure 1D). However, similar to the case for pupal development time, the diet type × fly group interaction effects were non-significant (*F*_8_,_285_ = 1.49; *p* = 0.15). We tested for differences in the rate of adult emergence. Adult eclosion was significantly affected by diet type (*F*_4_,_30_ = 55.27; *p* = 0.001) and gut bacterial manipulation (*F*_2_,_30_ = 75.19; *p* = 0.001). The diet type × fly group interactions were also significant (*F*_8_,_30_ = 2.52; *p* = 0.03). The adult eclosion rates of symbiotic and gnotobiotic larvae were significantly higher than that of axenic flies. A reduction in the protein content of the diet also reduced the adult eclosion rate compared to those for other diet types (Figure 1E).

### Effect of Diet and Gut Bacterial Manipulation on Immune Function and Survival of *B. dorsalis* Larvae

**FIGURE 2:**
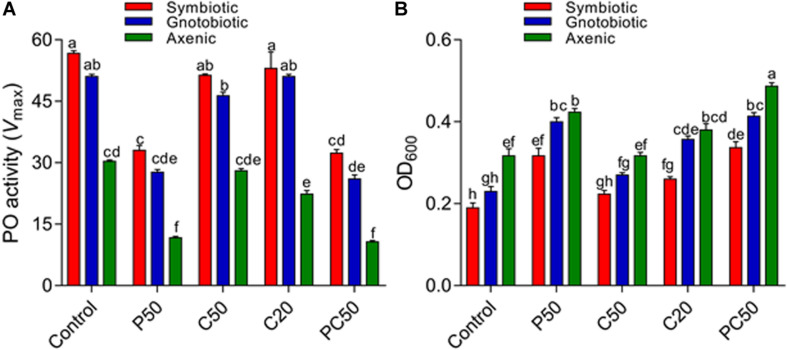
Effect of diet and gut bacterial manipulation on the immune function of *B. dorsalis* larvae: PO activity **(A)** and antibacterial activity **(B)**.

We analyzed hemolymph PO activity and antibacterial activity in the larvae fed on various diet and bacterial manipulation treatments. PO activity was significantly affected by bacterial manipulation (*F*_2_,_30_ = 548.66; *p* < 0.001) and diet type (*F*_4_,_30_ = 231.77; *p* = < 0.001). The interaction between diet type and fly groups was also significant (*F*_8_,_30_ = 5.03; *p* = < 0.001). PO activity was significantly reduced in larvae from axenic flies compared to larvae in gnotobiotic and symbiotic groups. The reintroduction of four bacterial isolates significantly increased PO activity; however, the PO activity was less than that of symbiotic flies. The reduction in carbohydrates and protein contents in the diet affected PO activity differently. PO activity was significantly lower in the hemolymph of larvae reared on the PC50 or P50 diet, followed by C50 and C20, compared to the control diet (Figure 2A). The antibacterial activities of hemolymph collected from different treatment groups differed. Hemolymph collected from larvae of the symbiotic group showed higher antibacterial activity than the other two groups (*F*_2_,_30_ = 134.52; *p* < 0.01). The reintroduction of bacterial isolates significantly affected the antibacterial activity, but similar to the PO activity, the activity was less than that of the symbiotic group. Hemolymph collected from larvae reared on a reduced protein content diet (PC50, P50) showed the less antibacterial activity than those reared on the C20, C50, or control diet (*F*_4_,_30_ = 112.37; *p* < 0.01; Figure 2B). A marginally significant interaction between bacterial manipulation and diet type (*F*_8_,_30_ = 2.35; *p* = 0.05) on the antibacterial activity of hemolymph was also observed.

**FIGURE 3:**
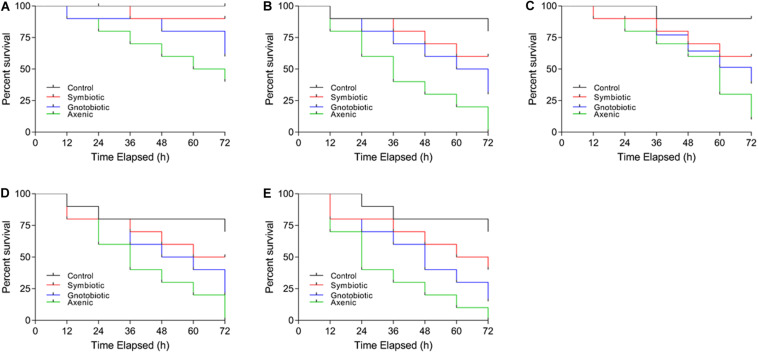
Effect of diet and gut bacterial manipulation on the survival of *B. dorsalis* larvae after infection: control **(A)**, P50 **(B)**, C50 **(C)**, C20 **(D)**, and PC50 **(E)**.

The post-infection survival of larvae infected with *E. coli* and *S. aureus* was calculated. The survival rates of larvae from different treatment groups differed (Figures 3A–E). The lowest survival was observed in larvae from the axenic groups fed the PC50 or P50 diet. The survival rates of gnotobiotic and axenic larvae feeding were not similar. The reduction in carbohydrate contents in the diet also affected the survival of larvae. Overall, reduced survival was observed in axenic group larvae feeding on a reduced protein content diet, with less reduction for the gnotobiotic larvae.

### Effect of Diet and Gut Bacterial Manipulation on Nutritional Indices of Hemolymph

**FIGURE 4:**
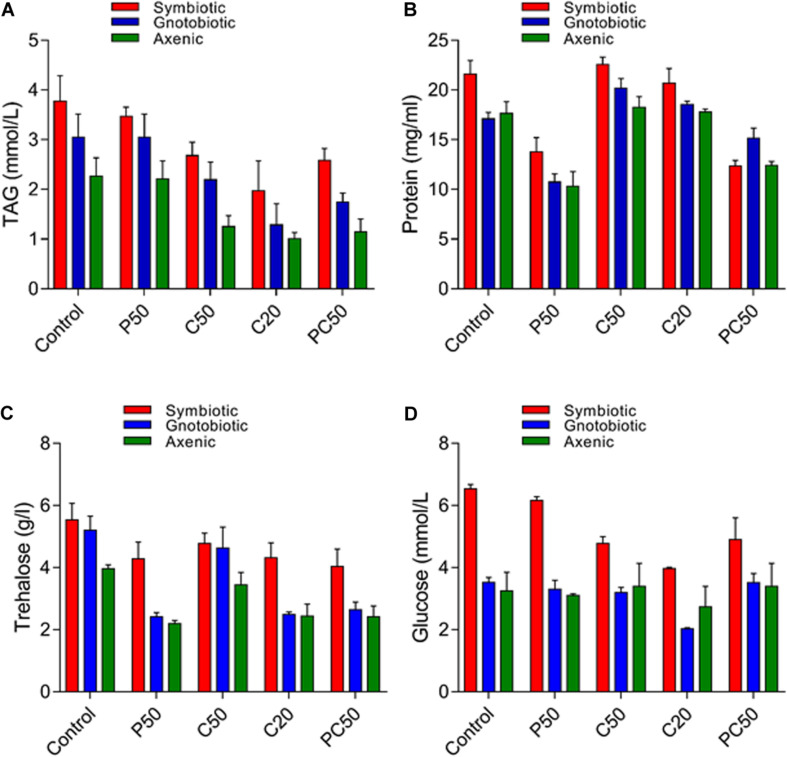
Nutritional indices in the hemolymph of *B. dorsalis* larvae feeding on different diets after bacterial manipulation: TAG **(A)**, protein **(B)**, trehalose **(C)**, and glucose **(D)**.

Significant differences were found in our assayed nutrient indices in hemolymph larvae from axenic, symbiotic, and gnotobiotic groups feeding on the different types of diets (Figures 4A–D). Bacterial manipulation had a significant effect on the TAG contents of larval hemolymph (*F*_2_,_30_ = 16.49; *p* < 0.001). However, a reduction in protein content in the diet (P50) did not affect the TAG content. Larvae fed a reduced carbohydrate diet (C50, C20, PC50) had reduced TAG content in the hemolymph (*F*_4_,_30_ = 11.14; *p* < 0.01). The interaction between diet type and the fly group was not significant (*F*_8_,_30_ = 0.18; *p* = 0.99). The protein content of the hemolymph was affected by bacterial manipulation (*F*_2_,_30_ = 0.30; *p* = 0.03), and a reduction in the protein content of the diet also significantly reduced the protein content in the hemolymph (*F*_4_,_30_ = 1.36; *p* = 0.04). The interaction between diet type and fly group was not significant (*F*_8_,_30_ = 0.02; *p* = 1.0). Trehalose contents in the larval hemolymph were significantly affected by bacterial manipulation (*F*_2_,_30_ = 22.95; *p* < 0.01) and diet type (*F*_4_,_30_ = 14.51; *p* < 0.01). The interaction diet type × fly group was not significant (*F*_8_,_30_ = 1.13; *p* = 0.37). Trehalose contents of hemolymph collected from larvae of axenic and gnotobiotic were similar. Thus, the reintroduction of bacterial isolates (gnotobiotic) did not increase the trehalose contents. The hemolymph of larvae fed C20, PC50, or P50 diets showed lower trehalose contents than that of larvae fed the C50 or control diet. Like trehalose, glucose contents in the hemolymph of gnotobiotic and axenic groups were similar (*F*_2_,_30_ = 42; *p* < 0.001). Compared to those fed the other diets, larvae fed the C20 or C50 diet showed a significant reduction in glucose contents in the hemolymph (*F*_4_,_30_ = 5.62; *p* = 0.002). The interaction between diet type and fly group was non-significant (*F*_8_,_30_ = 1.57; *p* = 0.17).

**FIGURE 5:**
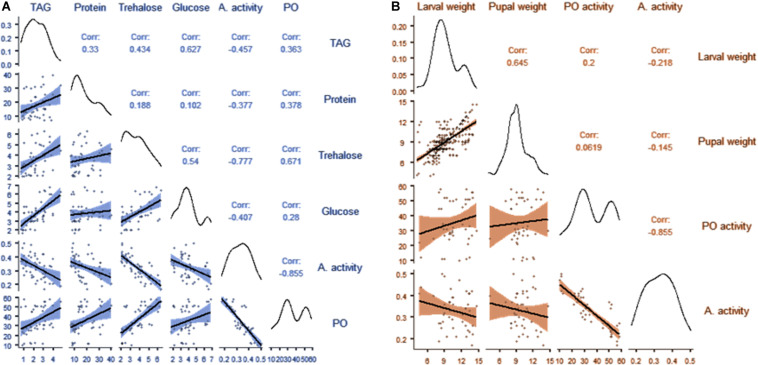
Correlation matrix showing the relationships between the nutritional indices and immunity of *B. dorsalis* larvae **(A)**; correlation matrix showing the relationships between the weight of larvae, the weight of pupae and immunity in *B. dorsalis* larvae **(B)**.

Correlation analysis between nutrient indices and the immunity of *B. dorsalis* larvae is presented in Figure 5A and Supplementary Table S2. The results showed a weak correlation between PO activity and glucose, TAG and protein content of the hemolymph (*r* < 4). However, we observed a moderate correlation between the trehalose content of the hemolymph and PO activity (*r* = 0.671; *p* < 0.001). No correlation was observed between the antibacterial activity of hemolymph and the nutritional indices of the hemolymph. Similarly, a weak correlation between either larval weight or pupal weight and PO activity was observed. However, there was no correlation between antibacterial activity and either larval or pupal weight (Figure 5B and Supplementary Table S3).

## Discussion

*Bactrocera dorsalis* harbors a complex gut microbiota with profound effects on behavior, mating competitiveness, pesticide degradation, and nutrition assimilation (Shi et al., 2012; Cheng et al., 2017; Akami et al., 2019; Khan et al., 2019). Previous studies indicated that some of the tephritid gut microbes, such as *Citrobacter freundii*, *Klebsiella*, *Providencia*, and other taxa belonging to Enterobacteriaceae, are transferred vertically to the next generation of the fly (Lauzon et al., 2009; Guo et al., 2017; Deutscher et al., 2018). However, little is known about whether specific vertically transmitted gut bacterial communities interact to shape insect development, fitness, and immunity along with diet macronutrients that mediate normal physiological functioning in insects.

In the current study, we demonstrated the combined effects of manipulating diet and the vertically transferred gut microbiota on the fitness and immunity of *B. dorsalis* larvae. The relationship between insects and their gut microbiota involves multiple interactions that vary with the composition of the microbiota and environmental factors, such as diet (Wong et al., 2014). Diet composition and bacterial manipulation both induced expected effects on the fitness traits of *B. dorsalis* larvae (Economopoulos et al., 1990; Kaspi et al., 2002; Chang and Cohen, 2009; Khaeso et al., 2018). Body mass and developmental times of larvae and pupa were significantly decreased and increased, respectively, in the larvae of the axenic group compared to those of the symbiotic and gnotobiotic groups. The reintroduction of the selected bacterial strain significantly improved the body mass and shortened the larval period compared to those of axenic flies, but the values were not similar to those of symbiotic flies. Unlike the larval development times, the pupal development times of axenic and gnotobiotic larvae were similar but longer than that of symbiotic larvae. Pupal weight was reduced in axenic flies compared to symbiotic and gnotobiotic flies. The reintroduction of bacteria in the flies increased the pupal weight. Similarly, the adult eclosion rate of symbiotic and gnotobiotic larvae was significantly higher than that of axenic flies.

*Klebsiella*, *Citrobacter*, *Providencia*, and *Enterobacter* species are the most abundantly represented species in *B. dorsalis* and belong to the family Enterobacteriaceae (Wang et al., 2011; Gujjar et al., 2017). These strains were selected for the current study based upon their mode of transmission, high abundance in the *B. dorsalis* gut, and role in flies according to the literature. A reduction in developmental time and an improvement in larval or pupal weight may have been caused by the symbiotic bacteria helping their hosts extend their nutritional range either by improving digestion efficiency or by providing digestive enzymes, vitamins or protein (Ben-Yosef et al., 2010; Wong et al., 2014; Kyritsis et al., 2019). Enterobacterial communities contribute to longevity, nitrogen and carbon metabolism, development, and copulatory success in some tephritid flies (Behar et al., 2008a,b). Previous studies showed that *Enterobacter* improved pupal and adult productivity and increased development by shortening the immature stages of *Ceratitis capitata* (Hamden et al., 2013; Augustinos et al., 2015). Similarly, Kyritsis et al. (2017, 2019) reported that consumption of *Enterobacter*- and *Klebsiella oxytoca*-based diets by *C. capitata* resulted in decreased immature stage mortality, reduced immature developmental duration, increased pupal weight, and prolonged survival by acting as the primary protein source. Previous studies showed that incorporation of *Klebsiella*, *Enterobacter*, and *Citrobacter* species into the diet improved the fitness of tephritid fly larvae (Hamden et al., 2013). In addition, the gut microbiota affects a range of host developmental and physiological processes (Sommer and Bäckhed, 2013). In the current study, axenic flies had poorer performance than gnotobiotic and symbiotic flies. Although the reintroduction of symbiotic bacteria significantly improved developmental traits, it did not result in flies comparable with symbiotic flies for some fitness parameters. We reintroduced only four bacterial strains, and strains other than these might be involved in the development of *B. dorsalis*. Moreover, the difference in the development of symbiotic and gnotobiotic flies can be due to the detrimental effects of antibiotic treatments on the host. Previous studies have shown that in addition to removing gut bacteria, antibiotics have deleterious effects on host physiology, including inhibiting mitochondrial gene expression, interrupting mitochondrial proteostasis, and increasing epithelial cell death (Morgun et al., 2015; Wang et al., 2015; Heys et al., 2018). However, this warrants further research.

Larvae of *B. dorsalis* responded differently to variation in nutritional conditions to maximize their development. Nutritional stress extends growth periods by changing energy allocations to somatic maintenance (Boggs, 2009). Overall, protein manipulation in the larval diet, more than carbohydrate manipulation, was the limiting factor for the development of *B. dorsalis* (Zucoloto, 1987; Nestel et al., 2004; Hou et al., 2020). A diet containing low protein increased the larval development time compared to that for diets in which sugar content was reduced. This agrees with previous studies in which compared with protein content changes in the diet, carbohydrate alteration had no significant effect on the larval development time of *C. capitata* (Plácido-Silva et al., 2005; Nash and Chapman, 2014). Carbohydrate content manipulation had a marginally significant effect on the time of larval development in our study.

Similarly, protein reduction in the diet had a significant effect on pupal development time and weight (Chan et al., 1990; Nash and Chapman, 2014; Hou et al., 2020), and carbohydrate manipulation had no significant effect on pupal development time and weight (Nestel et al., 2004). However, this does not agree with the results of previous studies showing that pupae from larvae fed a reduced sugar diet emerged earlier than those fed a regular diet (Kaspi et al., 2002). A reduction in the protein content of the diet also reduced the adult eclosion rate (Hou et al., 2020) compared to those for other diet types. Protein is the most necessary component in the diet of tephritid fruit flies, and its quantity and quality are essential for their proper development (Zucoloto, 1987; Chan et al., 1990).

Nutrition and gut microbes are critical to insect immune defense and resistance to pathogens (Cunningham-Rundles et al., 2005; Mason et al., 2019; Muhammad et al., 2019). The current study showed that deprivation of the gut microbiota drastically impaired the PO activity, antibacterial activity, and survival ability of *B. dorsalis* larvae after challenge with *E. coli* and *S. aureus*. The reintroduction of four bacterial isolates significantly enhanced the immunocompetence of *B. dorsalis* larvae; however, they were still less immunocompetent than the larvae of symbiotic flies, meaning that some other microbes or factors are involved. PO and antibacterial activities were significantly lower in the hemolymph of larvae reared on a diet with reduced protein contents (P50, PC50), followed by those for C50 and C20, compared to those for the control diet. The effects of dietary nutrients on the insect immune system are typically studied in terms of the macronutrient protein and carbohydrate and immune traits that are differentially affected by macronutrient intake (Povey et al., 2009; Srygley et al., 2009; Cotter et al., 2011). Our results confirm those findings on how the manipulation of nutrient contents reduces investments in the immune system (Alonso-Alvarez and Tella, 2001; Krams et al., 2015, 2017) while also reducing symbiont numbers and microbiota diversity. The positive association between gut microbes and the immune system shows that diet quality may result in symbiont involvement in the digestion of nutrients (David et al., 2014; Carmody et al., 2015; Krams et al., 2017).

Recent studies on the immunomodulatory impacts of commensal bacteria have identified that these effects are specific for individual bacteria, groups of bacteria, or specific components of the microbiota (Ivanov and Honda, 2012). The exact effect and mode of action of individual commensal bacteria are mostly unknown in *B. dorsalis* larvae. Here, we determined the stimulatory effects of the gut microbiota on *B. dorsalis* immunity by removing the entire commensal microbiota and reintroducing four bacterial strains. Therefore, the possible specific roles of individual bacteria or groups of bacteria on the immune system of *B. dorsalis* larvae deserve further investigation by making gnotobiotic larvae using individual strains. Mounting evidence has uncovered that the gut microbiota could inhibit the development of pathogens in insects by upregulating some important immune genes and that loss of specific bacterial components of the gut microbiota correlates with increased susceptibility to pathogenic infection in insects (Gonzalez-Ceron et al., 2003; Boissière et al., 2012; Bahia et al., 2014; Muhammad et al., 2019). Reintroduction of *Enterobacter* in the gut of the fall armyworm increased total hemocyte counts by 100% and PO activity by 140% in the hemolymph compared with those of axenic larvae (Mason et al., 2019). Similarly, *Enterobacter* species isolated from the guts of wild mosquitos conferred resistance to *Plasmodium* infection through ROS produced by the bacterium itself, rather than eliciting an immune response that reduces parasite load (Cirimotich et al., 2011). Bacteria from the endogenous microbiota of insects, including *Providencia rettgeri* and *Morganella morganii*, outcompete non-endogenous species, including *E. coli*, in the gut of insects and reduce the chances of their colonization, thus increasing the immunocompetence of the insects (Wang and Rozen, 2018).

Our results also revealed that the contents of hemolymph protein, glucose, trehalose, and TAGs in larvae from axenic flies were significantly lower than those in larvae of the symbiotic group after they fed on most of the tested diets. Reintroducing gut microbes increased the contents of protein and TAGs, showing only that some other bacterial species or factors are involved. Gut bacterial isolates are involved in the enhancement of dietary protein digestion and amino acid intake by upregulating the expression of intestinal peptidases (Erkosar et al., 2015). In *D. melanogaster*, it has been found that axenic flies have altered insulin signaling and lipid metabolism (Shin et al., 2011). Therefore, gut bacterial reintroduction could restore high nutrition indices (Habineza et al., 2019). This further suggests that the decreased body mass gain of larvae from the axenic group is the effect of the gut microbiota absence causing nutritional metabolic defects (Shin et al., 2011; Storelli et al., 2011, 2018; Habineza et al., 2019). Some of the tested bacterial strains have also been involved in the production or metabolism of protein. Collectively, the mechanisms behind the introduction of bacteria and diet type affecting the nutrient metabolism of *B. dorsalis* larvae need to be defined further.

## Conclusion

In summary, our results showed that the tested vertically transmitted bacterial isolates and diet interactions significantly affect the fitness and immunity of *B. dorsalis*. Larvae of axenic flies grew slower and displayed weaker immune-based responses than larvae of gnotobiotic and symbiotic flies. Moreover, we also found the significant effect of protein level in the diet on fitness of *B. dorsalis*. These findings are also a good illustration of possibilities that the vertically transmitted gut bacteria can be used for the improvement of insect mass production in support of SIT applications.

## Data Availability Statement

Sequence data generated in this study has been deposited at NCBI under Bio projects PRJNA665789 and PRJNA357667.

## Author Contributions

BH and YX: conceptualization and methodology. BH: formal analysis. BH and JS: investigation, data curation, and writing – original draft preparation. YX: resources, writing – review and editing, visualization, supervision, project administration, and funding acquisition. All authors have read and agreed to the published version of the manuscript.

## Conflict of Interest

The authors declare that the research was conducted in the absence of any commercial or financial relationships that could be construed as a potential conflict of interest.

## Footnotes

1 https://www.genewiz.com.cn/

## Abbreviations

PO: Phenoloxidase
PBS: Phosphate-buffered saline
OD: Optical density
AMP: Antimicrobial peptide
CFU: Colony-forming unit
LB: Luria-Bertani
ROS: Reactive oxygen species
PTU: Phenylthiourea
TAG: Triglyceride
GLM: General linear model.
