# Supplementary material for: Vertically Transmitted Gut Bacteria and Nutrition Influence the Immunity and Fitness of Bactrocera dorsalis Larvae
Source: Front Microbiol. 2020 Oct 30;11:596352. doi: 10.3389/fmicb.2020.596352 (PMC7661685; doi:10.3389/fmicb.2020.596352)
Supplement: Supplementary file 1 [file Data_Sheet_1.docx]

Supplementary Material

**Supplementary Tables**

**Table S1**. Identity of gut bacterial isolates from *Bactrocera dorsalis* adult flies based on 16S rRNA gene sequences

| **Isolates** | **Sequence length (bp)** | **Best BLAST hit species** | **GenBank accession no.** | **Query cover (%)** | **Identity (%)** |
| --- | --- | --- | --- | --- | --- |
| Citrobacter sp. | 1521 | *Citrobacter* sp. CF-BD | KR002082 | 100 | 100 |
| Klebsiella sp. | 1106 | *Klebsiella pneumoniae* | CP054063.1 | 99 | 96.26 |
| Enterobacter sp. | 1128 | *Enterobacter* sp. strain AD2-1 | MN400346.1 | 99 | 97.18 |
| Providencia sp. | 1168 | *Providencia rettgeri* strain P6 | MK049957.1 | 99 | 99.16 |

| **Table S2** Correlation matrix showing the relationships between the nutritional indices and immunity of *B. dorsalis* larvae | | | | | | | | | | | | | | | | |
| --- | --- | --- | --- | --- | --- | --- | --- | --- | --- | --- | --- | --- | --- | --- | --- | --- |
|  |  |  |  |  |  |  |  |  |  |  |  |  |  |  |  |  |
|  | |  | | **TAG** | | **Protein** | | **Trehalose** | | **Glucose** | | **Antibacterial activity** | | **PO activity** | | |
| **TAG** |  | Pearson's r |  | — |  |  |  |  |  |  |  |  |  |  |  |  |
|  |  | p-value |  | — |  |  |  |  |  |  |  |  |  |  |  |  |
| **Protein** |  | Pearson's r |  | 0.330 |  | — |  |  |  |  |  |  |  |  |  |  |
|  |  | p-value |  | 0.027 |  | — |  |  |  |  |  |  |  |  |  |  |
| **Trehalose** |  | Pearson's r |  | 0.434 |  | 0.188 |  | — |  |  |  |  |  |  |  |  |
|  |  | p-value |  | 0.003 |  | 0.215 |  | — |  |  |  |  |  |  |  |  |
| **Glucose** |  | Pearson's r |  | 0.627 |  | 0.102 |  | 0.540 |  | — |  |  |  |  |  |  |
|  |  | p-value |  | < .001 |  | 0.504 |  | < .001 |  | — |  |  |  |  |  |  |
| **Antibacterial activity** |  | Pearson's r |  | -0.457 |  | -0.377 |  | -0.777 |  | -0.407 |  | — |  |  |  |  |
|  |  | p-value |  | 0.002 |  | 0.011 |  | < .001 |  | 0.006 |  | — |  |  |  |  |
| **PO activity** |  | Pearson's r |  | 0.363 |  | 0.378 |  | 0.671 |  | 0.280 |  | -0.855 |  | — |  |  |
|  |  | p-value |  | 0.014 |  | 0.010 |  | < .001 |  | 0.063 |  | < .001 |  | — |  |  |
|  | | | | | | | | | | | | | | | | |

| **Table S3**. Correlation matrix showing the relationships between the weight of larvae, the weight of pupae and immunity in *B. dorsalis* larvae | | | | | | | | | | | | |
| --- | --- | --- | --- | --- | --- | --- | --- | --- | --- | --- | --- | --- |
|  |  | | | **Larval weight** | | **Pupal weight** | | **PO activity** | | **Antibacterial activity** | |  |
| Larval weight |  | Pearson's r |  | — |  |  |  |  |  |  |  |  |
|  |  | p-value |  | — |  |  |  |  |  |  |  |  |
| Pupal weight |  | Pearson's r |  | 0.645 |  | — |  |  |  |  |  |  |
|  |  | p-value |  | < .001 |  | — |  |  |  |  |  |  |
| PO activity |  | Pearson's r |  | 0.200 |  | 0.062 |  | — |  |  |  |  |
|  |  | p-value |  | 0.187 |  | 0.686 |  | — |  |  |  |  |
| Antibacterial activity |  | Pearson's r |  | -0.218 |  | -0.145 |  | -0.855 |  | — |  |  |
|  |  | p-value |  | 0.151 |  | 0.340 |  | < .001 |  | — |  |  |
|  | | | | | | | | | | | | |

**Supplementary Figures**
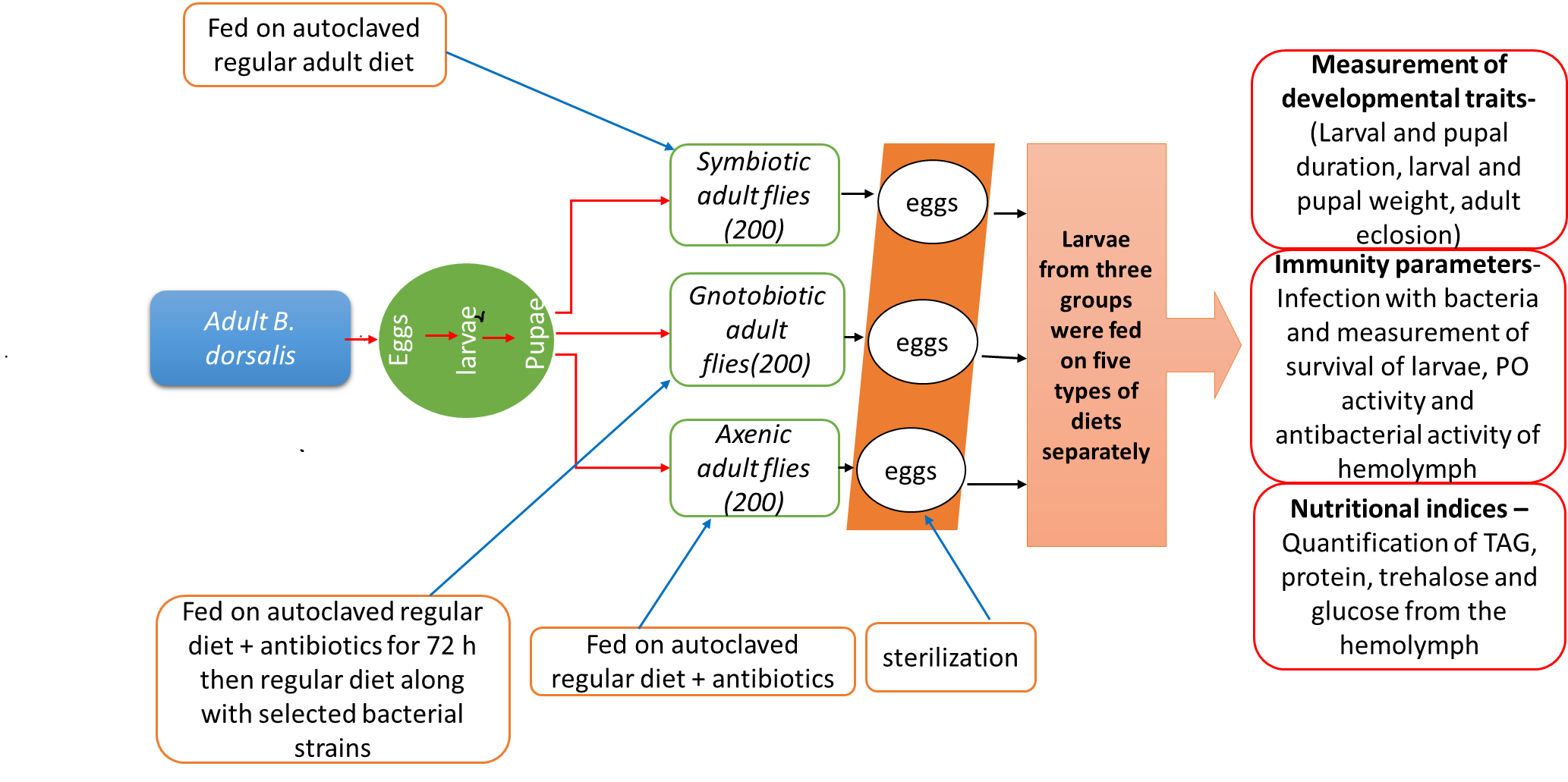


**Fig. S1:** Schematic representation of experiments

**(a)**

Gnotobiotic


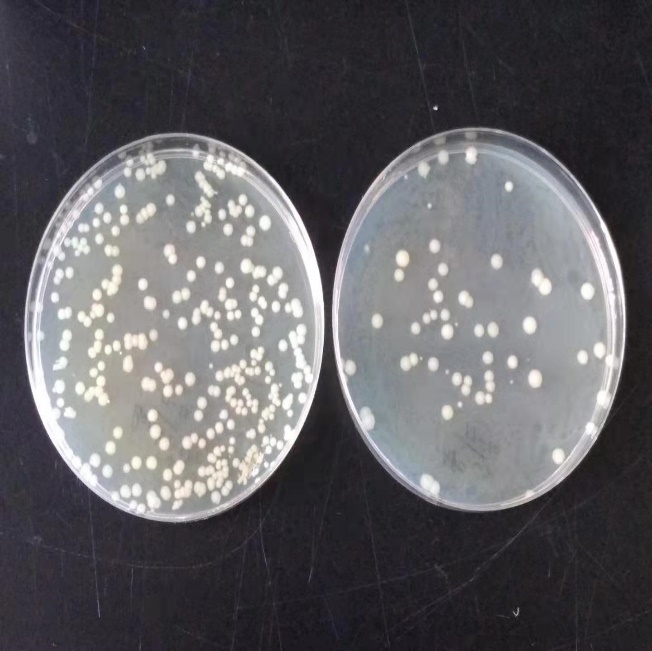

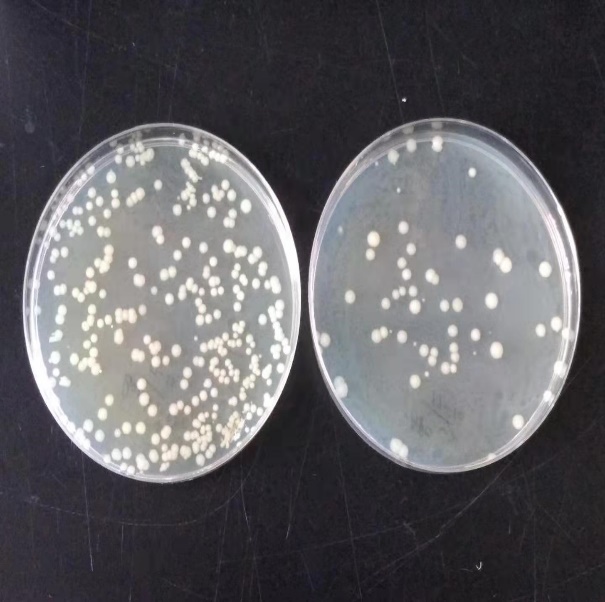

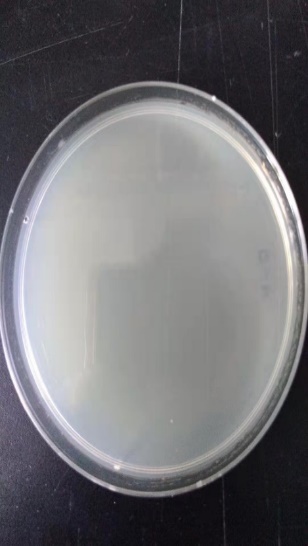


Symbiotic

Axenic


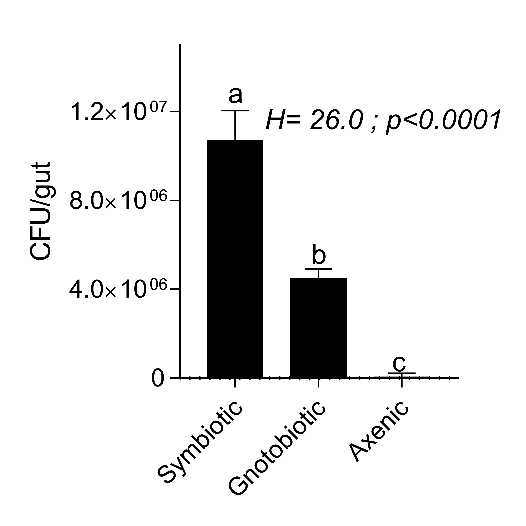

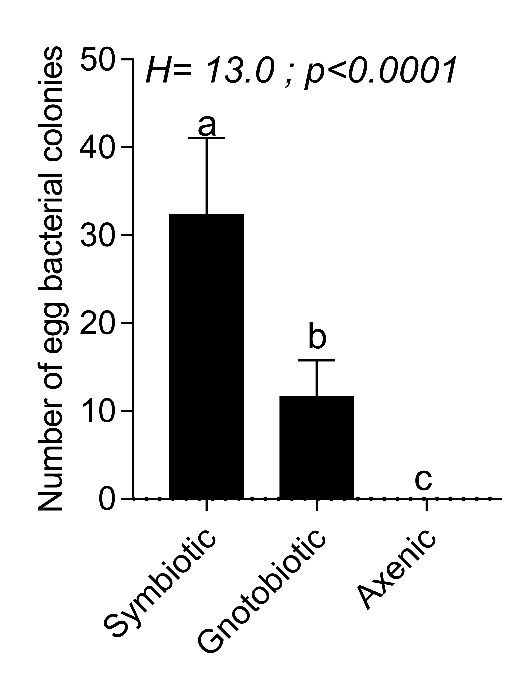


**(b)**

**(c)**

**Figure S2**: Number of bacterial colonies observed in three groups of flies before egg collection (a, b), detection of bacteria from newly laid eggs of *B. dorsalis*


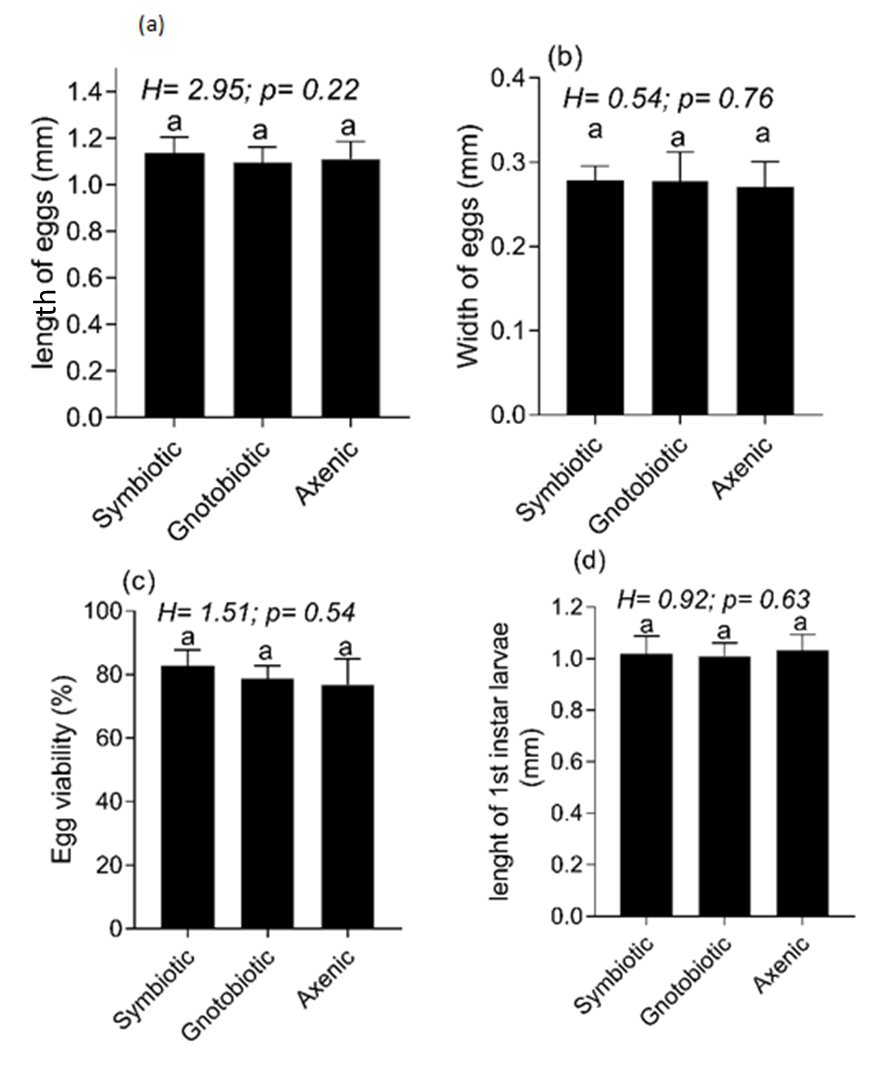


**Figure S3**. Length (a) and width of eggs (b) collected from three groups of flies, egg hatchability (%) (c), and length of newly emerged larvae (d)
